# Draft Genome Sequences of Two *Kocuria* Isolates, *K. salsicia* G1 and *K. rhizophila* G2, Isolated from a Slaughterhouse in Denmark

**DOI:** 10.1128/genomeA.00075-16

**Published:** 2016-03-31

**Authors:** Jakob Herschend, Prem K. Raghupathi, Henriette L. Røder, Søren J. Sørensen, Mette Burmølle

**Affiliations:** Section for Microbiology, Department of Biology, University of Copenhagen, Copenhagen, Denmark

## Abstract

We report here the draft genome sequences of *Kocuria salsicia* G1 and *Kocuria rhizophila* G2, which were isolated from a meat chopper at a small slaughterhouse in Denmark. The two annotated genomes are 2.99 Mb and 2.88 Mb in size, respectively.

## GENOME ANNOUNCEMENT

*Kocuria rhizophila* and *Kocuria salsicia* are Gram-positive, coccoid, spherical saprotrophic bacteria belonging to the family *Micrococcineae*. *Kocuria* species are ubiquitous and highly adapted to their ecological niches (1) and are mainly identified in soil samples (2), clinical specimens (3, 4), fermented food (5, 6), and as members of the oral and skin flora (7). *K. rhizophila* is also commonly used as a standard quality control strain for antimicrobial susceptibility testing (2). Currently, there is one complete genome and one draft genome sequence publicly available of *K. rhizophila*: *K. rhizophila* DC2201 (2) and *K. rhizophila* P7-4 (1). Here, we present the draft assembly of *K. salsicia* G1 and *K. rhizophila* G2, isolated from a slaughterhouse in Denmark (8).

The whole-genome sequencing libraries were prepared using the Nextera XT kit (Illumina, USA), according to the manufacturer’s recommendations, and then sequenced as part of the flow cell, as 2 × 250-base paired-end reads using the Illumina MiSeq (Illumina) technology. The reads were cleaned and trimmed using CLC Genomics Workbench 7 (CLC bio, Denmark). Quality-filtered reads were assembled using SPAdes version 3.5.0 (9). The annotations on the resulting contigs were performed on the RAST server (10) and RNAmmer 1.2 (11) to check and screen for noncoding RNAs.

The assembly of *K. salsicia* G1 resulted in 199 contigs at 27× coverage, with an average G+C content of 70.43%. *K. rhizophila* G2 is assembled into 87 contigs at 126× coverage, with an average G+C content of 70.81%. The annotated results from G1 predicted 2,565 coding sequences, with an average length of 971 bp (1,172 coding sequences [CDSs] have functional predictions), 19 tRNA-coding genes, and 5 rRNA-coding genes. The predictions from G2 included 2,531 coding sequences, with an average length of 955 bp (1,154 CDSs have functional predictions), 18 tRNA-coding genes, and 7 rRNA-coding genes. Both strains had single predicted copies of 16S and 23S rRNA genes, with the only difference in 5S rRNA gene copies, with 3 for G1 and 5 for G2. There are 359 and 358 predicted subsystems in the genomes of G1 and G2, respectively. Metabolic network comparisons revealed 1,774 putative protein-encoding genes (PEGs) conserved in both G1 and G2 genomes. In a function-based comparison to the genome of DC2201, the genomes of G1 had 179 unique PEGs and 147 PEGs in G2. The main differences observed in a comparison of *K. salsicia* G1 to *K. rhizophila* DC2201 and *K. rhizophila* G2 were the presence of sequences encoding clustered regularly interspaced short palindromic repeat (CRISPR) elements, iron acquisition, and metabolism subsystems identified in G1 only. These suggest a prominent influence of phage exposure and possible adaptation mechanisms of isolate G1 to a more densely populated environment, such as the animal gut. Further work with these genomes is expected to facilitate the identification and understanding of genes associated with adaptive mechanisms of these strains and biofilm formation.

### Nucleotide sequence accession numbers.

The whole-genome sequencing (WGS) projects for *K. salsicia* G1 and *K. rhizophila* G2 have been deposited at the European Nucleotide Archive (ENA) under the contig accession numbers CZJU01000001 to CZJU01000199 and CZJW01000001 to CZJW01000087, respectively. The versions described in this paper are the first versions.
